# Bibliometric Analysis of the 100 Most Cited Systematic Reviews and Meta-Analysis in Oral Pathology

**DOI:** 10.7759/cureus.91013

**Published:** 2025-08-26

**Authors:** Pillai Arun Gopinathan, Ali Aboalela, Ikram UI Haq, Bijesh Yadav, Salman Siddeeqh, Rajkiran Chitumalla, Sulthan Ibrahim R Khan, Abdulah Mohammed Alyahya, Faris Fahad Alanazi, Abdulrahman Khalid Alwuayl, Abdullah Sami Almedlej, Nasser Mohammed Alqarni

**Affiliations:** 1 Department of Maxillofacial Surgery and Diagnostic Sciences, College of Dentistry, King Saud Bin Abdulaziz University for Health Sciences, King Abdullah International Medical Research Center, Ministry of National Guard Health Affairs, Riyadh, SAU; 2 Department of Dentistry, College of Dentistry, King Saud Bin Abdulaziz University for Health Sciences, Riyadh, SAU; 3 Department of Population Health, King Abdullah International Medical Research Center, Riyadh, SAU; 4 Department of Restorative and Prosthetic Dental Sciences, College of Dentistry, King Saud Bin Abdulaziz University for Health Sciences, King Abdullah International Medical Research Center, Ministry of National Guard Health Affairs, Riyadh, SAU; 5 Dentistry, National Guard Health Affairs, Riyadh, SAU

**Keywords:** bibliometrics, citation analysis, meta-analysis, oral medicine, oral pathology, systematic reviews

## Abstract

The bibliometric study aimed to analyze the 100 most cited systematic reviews (SRs) and meta-analyses (MAs) in the field of oral pathology. This is the first research on SRs and MAs conducted on citation analysis pertaining to oral pathology. On December 24, 2024, a search was made in the Web of Science database for 3,000 articles. Out of that pool, the 100 most cited oral pathology-associated articles were selected after confirming their indexing status in the PubMed database. Standard information on the periodic growth, authors, affiliated organizations, publication year, citation metrics, and research topic domains was recorded. Statistical analysis was conducted via the Pearson Chi-square test and one-way analysis of variance (ANOVA). The VOSviewer program was employed to conduct a network analysis for co-occurring key terms. The results depicted the 100 most cited articles from subjects of oral pathology/oral medicine and associated head and neck pathology published between 1990 and 2022. The journal with the highest number of articles was *Oral Surgery, Oral Medicine, Oral Pathology, and Oral Radiology*, followed by *Oral Oncology*. Head/neck cancer and oral cancer were the most predominant research domains. The VOSviewer software revealed five interconnected clusters with 17 common keywords. Significant relationships were observed between citation impact and collaboration patterns among country publications and institutions (P < 0.001). Articles published under the head and neck pathology specialty, SR and MA article types, and non-dental journals had a higher citation impact and were statistically significant (P < 0.001). There has been a rise in the quantity of publications that are conducted in the field of oral pathology with respect to SRs and MAs. By referring to the most cited articles, this review could facilitate data-driven decision-making, improve research visibility, and influence the strategic direction for researchers and practitioners in oral and head/neck specialties.

## Introduction and background

Bibliometric analysis is a scientific process that uses computer assistance to systematically analyse and identify fundamental research features by encompassing all publications pertinent to a specific topic or field. It generally quantifies research outputs such as publication frequencies, citation frequencies, and metrics by generally keeping a track of how many times the article is cited after it has been published [1]. Citation analysis, a conventional bibliometric method, quantifies the quantity and interrelation of references a publication accumulates over time [2]. It additionally emphasises the advancement of science and the calibre of research conducted over the years, as well as the focal areas of ongoing research. Although it is a time-dependent metric, it signifies the influence and advancement of research over the years and serves as an often-utilised indication of scientific excellence [3].

The evidence-based medicine pyramid indicates that systematic reviews (SRs) and meta-analyses (MAs) represent the greatest degree of evidence, as they consolidate validated information from diverse existing literature [4]. SRs are structured to address specific enquiries by utilising a defined technique to thoroughly search for, select, evaluate, and analyse original research articles. SRs may include official MAs or may not. MA is the statistical aggregation of findings from studies included in an SR, offering a substantial benefit to SRs by augmenting the overall sample size through the amalgamation of data from individual studies, thereby enhancing the statistical power and precision in evaluating treatment effects [5].

Bibliometric analysis in the field of oral pathology has been conducted on topics such as odontogenic cysts and tumours [6], oral vesiculobullous lesions [7], oral proliferative verrucous [8], and leukoplakias and oral cancer research [9]. Following the millennial transition, there was a significant surge in the quantity of published SRs in medicine and dentistry, particularly within the domain of oral pathology. Nevertheless, citation analysis of level one evidence comprising SRs and MAs has not been conducted in the domain of oral pathology exclusively. The primary objective of the review was to identify and analyse the top-cited SRs and MAs in oral pathology, with a focus on their citation trends, thematic patterns, and indexing status. By highlighting the most influential SRs/MAs, the study provides researchers, educators, and clinicians with a clearer understanding of the key areas that have shaped evidence-based knowledge in the field. This will help the scientific community to recognise research gaps, prioritise high-impact themes for future investigations, and use well-cited evidence as a foundation for clinical decision-making and policy formulation. Therefore, the present review aimed to identify and analyse the bibliometric characteristics of the 100 most-cited SR and MA in oral pathology.

The objectives of the review are as follows: 1) to assess comprehensive research growth and contribution in SRs and MAs in oral pathology; 2) to ascertain the most prolific country, journal, authors, and research organisations and examine various research domains for average citations per article; and 3) to recognise the co-occurrence network among authors and common keywords using the VOSviewer software.

## Review

Methodology

Ethical Considerations

The Institutional Review Board of the King Abdullah International Medical Research Centre sanctioned the study, as denoted by reference number NRR24/120/12. As it involved a retrospective analysis of data, ethical approval was exempted.

Search Criteria

**Figure 1:**
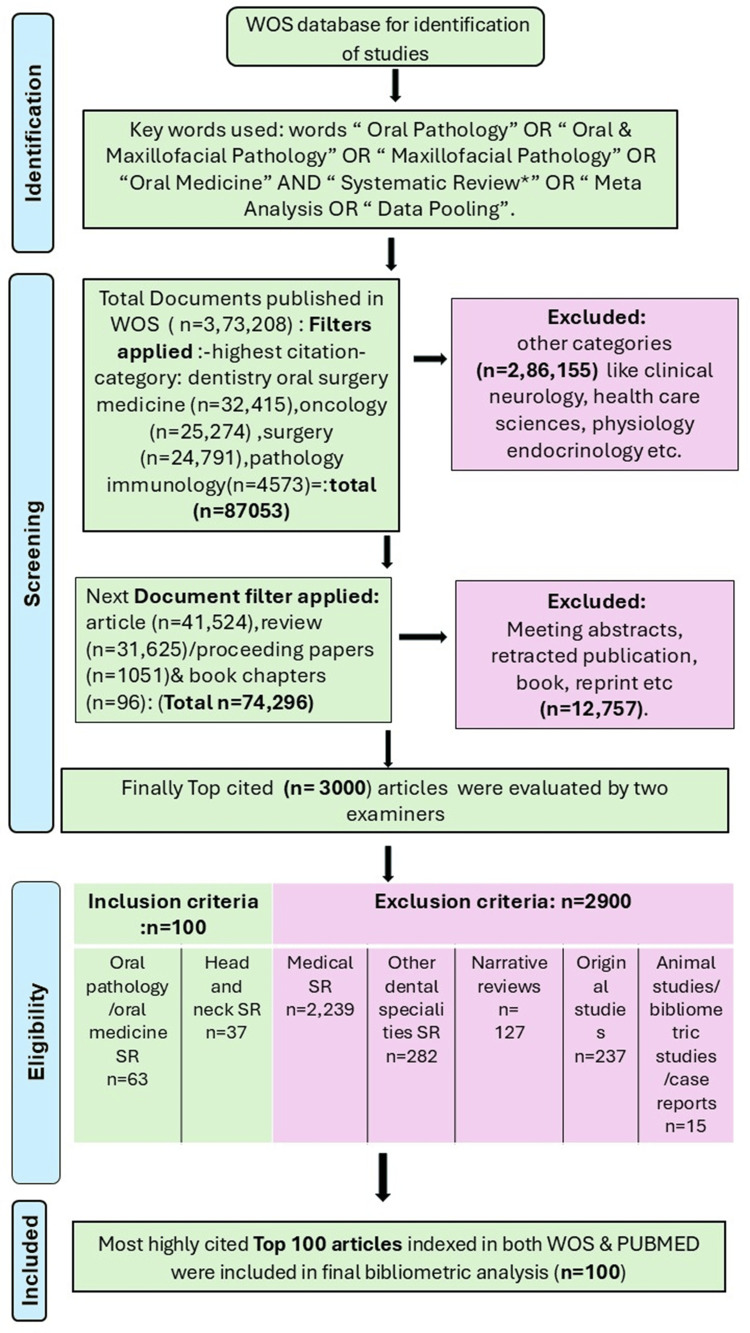
Criteria for the selection of the 100 most cited articles WoS: Web of Science

A search was performed in the core collection of Web of Science (WoS), due to its extensive thematic coverage and the capability to quantify article citations. The WoS database was searched on December 24, 2024, to identify the top-cited SRs and MAs in oral pathology. In the first week of January 2025, two of our team members retrieved and verified these articles from PubMed. Accordingly, the data collection process spanned from December 24, 2024, to January 7, 2025. As the analysis focused exclusively on the most cited SRs and MAs, studies published in 2023 and later were not included, as their recent publication dates resulted in relatively low citation counts. The search technique was executed in the subject area (under all fields) with the following keywords: “Oral Pathology” OR “Oral and Maxillofacial Pathology” OR “Maxillofacial Pathology” OR “Oral Medicine” AND “Systematic Review*” OR “Meta Analysis” OR “Data Pooling”. Initial search revealed n = 373,208 articles. Later, filters of dentistry, oral surgery, medicine, oncology, and surgery were added, which brought down the count to n = 87,053 articles. Finally, document filters of articles, review articles, proceeding papers, and book chapters were added, giving a total count of n = 73,201. Figure 1 shows the methodology of the flow chart. The excluded documents were meeting abstracts, letters, editorial materials, notes, early access, corrections, book reviews, retracted publications, meetings, news items, discussions, reprints, etc.

A total of 3,000 top-cited articles from WoS were recruited to a Microsoft Excel sheet (Microsoft Corp., USA). Two authors (AP and SS) conducted an independent preliminary screening of papers to evaluate their relevance to the study, utilising information from the title, abstract, and, when necessary, the whole article. Any disagreement over the inclusion of a certain article was settled by consensus discussion among all authors. The 100 top-cited articles indexed in both WoS and PubMed were selected for data extraction and further analysis. The primary source for identifying SRs/MAs was the Web of Science. The indexation status was further verified in PubMed, and any SR/MA not indexed in PubMed was excluded. The top 100 most-cited SRs/MAs were selected because citation count is a widely accepted indicator of scientific impact and influence. Focusing on the top 100 provided a manageable dataset while still capturing the most impactful contributions in oral pathology. This threshold has also been commonly used in bibliometric analyses, allowing comparison with previous studies and ensuring that only the most influential literature shaping the field was included.

Inclusion Criteria

SR and MA articles relevant to head and neck studies (which included oral cavity), as oral pathology and oral medicine are related specialities, both were considered. The article had to be indexed in both WoS and the PubMed database.

Exclusion Criteria

SRs and MAs on medical-related topics and other dental systematic reviews on specialities like dental materials, periodontics, prosthodontics, restorative, endodontics, pedodontics, oral surgery, orthodontics, and dental public health were excluded. Animal studies, narrative reviews, original studies, scoping reviews, case reports, case series, non-English articles and bibliometric studies were also excluded (Figure 1 shows the flow chart of the methodology).

Data Analysis and Statistical Analysis

The following variables, like periodic distribution of articles, total citations, journal listings, leading authors, prominent institutions, top countries, keyword analysis, prominent thematic areas, and characteristics of the 100 most cited articles, were evaluated. The bibliometric program VOSviewer (Leiden University, Leiden, Netherlands), version 1.6.10, was used. The country of publication was determined by considering the affiliations of all authors, following common practice in bibliometric studies. The dataset was downloaded into an Excel sheet, and the country and institutional affiliation for each author were included. For articles co-authored by authors from multiple countries, the publication was counted for every country and institution represented, ensuring that all contributing affiliations were included. The one-way ANOVA was used to evaluate the citation impact among countries and institutions, while the Pearson chi-square test was utilised to assess the citation impact among types of review articles, subject specialities, and journal types. R 4.4 software was used for the analysis (R Foundation for Statistical Computing, Vienna, Austria).

Results

Periodic Distribution of Articles

**Figure 2:**
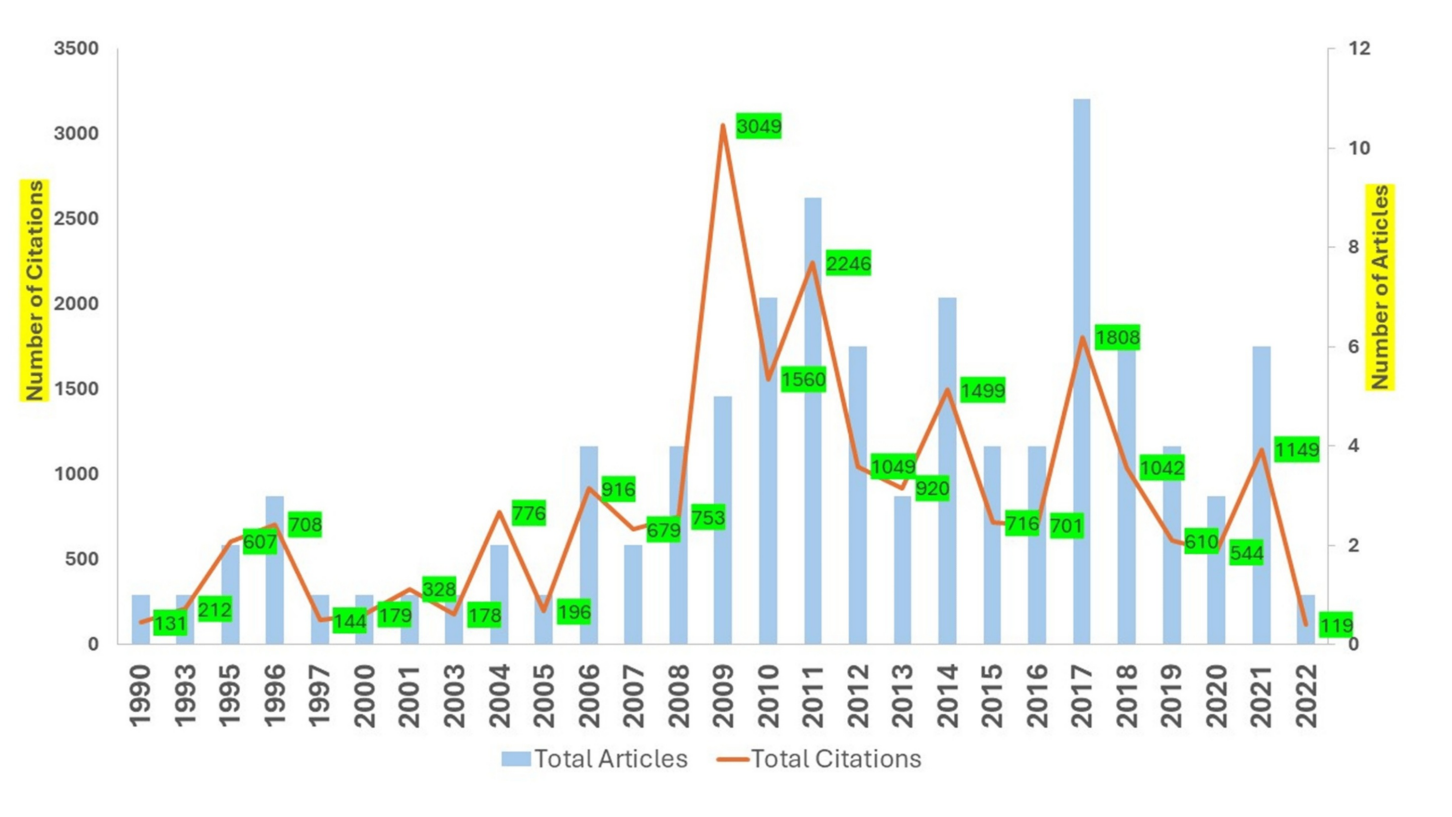
Year-wise distribution of the most cited articles and citations

Among the top 100 cited articles, the period of distribution of articles was published in a span ranging from 1990 to 2022. Figure 2 illustrates the chronological distribution of articles. The year 2017 recorded the most publications (n = 11), followed by 2011 (n = 9), while the years 2010 and 2014 had seven publications each. Most of the cited articles (n = 66) were published between 2010 and 2019. The article distribution was segmented into four segments, each encompassing around seven years. The third segment (2013-2020) yielded the highest number of published papers (n = 45), followed by the fourth segment period (2016-2022) with 35 articles. The first segment (1990-1997) had five articles, and the second segment (2000-2007) had seven articles. The top 100 articles received a total of 22,819 citations, and the average citation impact per article was about 228.39. The highest citation impact was attained by five articles published in 2009, with 609.8 citations per article, followed by two articles from 2004 and 2007, averaging 388 and 339.5 citations per article, respectively.

Premium Publication Sources

A total of 52 articles were included in the top 10 journals, which published three and more than three articles, with most of the journals belonging to the Q1 category (Table 1 shows the distribution of the top 10 journals with quartiles and cite scores). Around 70% of the articles were published in the six leading journals (n = 37), with the greatest number of articles published in the *Oral Surgery, Oral Medicine, Oral Pathology, and Oral Radiology* journal. The *Oral Surgery, Oral Medicine, Oral Pathology, and Oral Radiology* and *Radiotherapy and Oncology* journals together excelled significantly in terms of total articles (n = 14) and total citations (5,600). These publications had substantial output and the greatest citation count, reflecting their esteemed status in the domain of SR in oral pathology. As far as citation impact was considered, the *Radiotherapy and Oncology* journal was the highest, followed by the *International Journal of Cancer*. According to the citation metrics, Scopus cite score, WoS JIF (journal impact factor), and H-index (Scimago journal rank), the *Annals of Oncology* received the most metrics.

**Table 1:** Distribution of the top 10 journals

Serial no.	Journal name	JIF WoS (Quartile)	CiteScore - Scopus (Quartile)	SJR (H-Index)	Total articles	Total citations	Citation impact
1	Oral Surgery, Oral Medicine, Oral Pathology, and Oral Radiology	2 (Q2)	3.8 (Q1)	0.56 (139)	9	2190	243.33
2	Oral Oncology	4 (Q1)	8.7 (Q1)	1.26 (137)	7	1178	168.29
3	British Journal of Cancer	6.4 (Q1)	15.1 (Q1)	3 (272)	6	972	162.00
4	Head and Neck - Journal for the Sciences and Specialties of the Head and Neck	2.4 (Q1)	7 (Q1)	1.03 (147)	5	1397	279.40
5	Oral Diseases	2.9 (Q1)	7.6 (Q1)	1.09 (104)	5	863	172.60
6	Radiotherapy and Oncology	4.9 (Q1)	10.3 (Q1)	1.7 (182)	5	3410	682.00
7	International Journal of Cancer	5.7 (Q1)	13.4 (Q1)	2.13 (260)	4	1134	283.50
8	Journal of Dental Research	5.7 (Q1)	15.3 (Q1)	1.91 (211)	4	603	150.75
9	Journal of Oral Pathology & Medicine	2.7 (Q1	5.9 (Q1)	0.72 (95)	4	951	237.75
10	Annals of Oncology	56.7 (Q1)	63.9 (Q1)	13.94 (294)	3	456	152.00

Most Productive Countries, Institutions, and Authors

**Figure 3:**
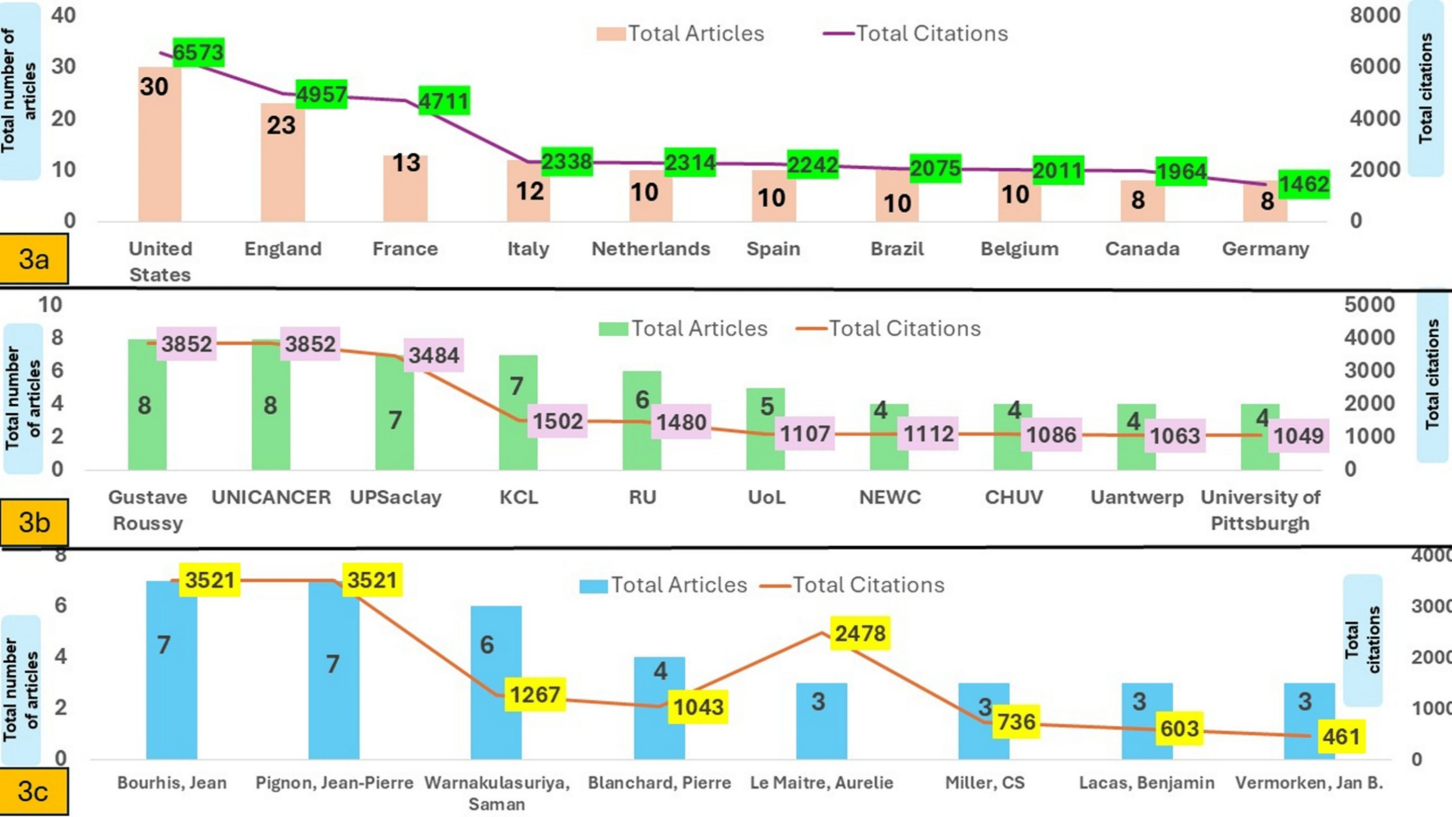
a) Top 10 most prolific nations illustrating the distribution of total citations and total articles. b) Top 10 institutions illustrating the distribution of total citations and total articles. c) Top authors illustrating the distribution of total citations and total articles. UPSaclay: Universite Paris Saclay, KCL: King College London, RU: Radboud University Nijmegen, UoL: University of London, CHUV: Centre Hospitalier Universitaire Vaudois, NEWC: Newcastle University – UK, UAntwerp: University of Antwerp

A total of 41 countries contributed to the 100 most cited articles. Authors from 13 countries produced one article each, while authors from nine countries contributed between six, three, and two articles each. The top 10 countries are shown in Figure 3a, which contributed more than seven articles each. The United States was the most productive nation in terms of total number of articles and total citations, while the highest citation impact was contributed by France.

A total of 276 institutions were identified, with 203 institutions contributing one article each, 43 institutions producing two articles each, and 16 institutions producing three articles each. The top 10 institutions are shown in Figure 3b, which are ranked according to research output and its impact. Notably, authors affiliated with Gustave Roussy and UNICANCER co-authored eight articles each, securing the top rank with the highest number of total articles and total citations. However, the Universite Paris Saclay received the highest citation impact.

A cumulative sum of 635 authors contributed to the 100 most cited articles, with 83.62% (n = 531) of authors contributing to a single article. Thirty-four authors contributed to two articles each, while the top eight authors contributed to more than two articles each. Figure 3c shows the distribution of top authors. Notably, Jean and Bourhis and Jean-Pierre and Pignon co-authored seven articles together, which accounts for their identical number of articles and citations. Le Maitre and Aurelie received the highest citation impact with three articles.

**Figure 4:**
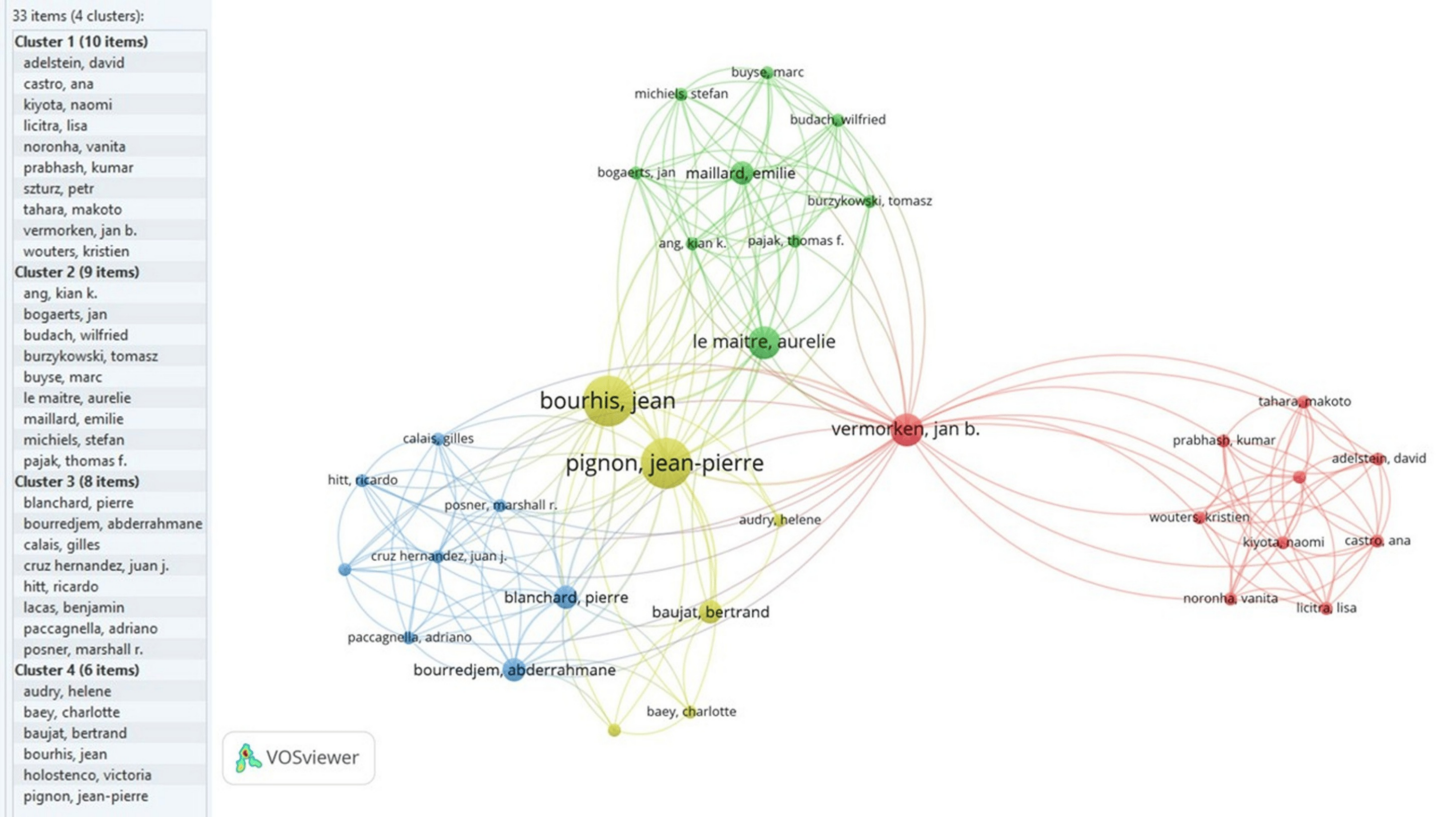
Co-occurrence network of authors

Of the total authors, only 33 authors in the VOSviewer network visualisation were connected through four clusters, as shown in Figure 4. The size of the nodes indicates the impact and connectivity in the dataset. Borghis, Jean, Pignon, and Jean Pierre were the most influential, while Vermorken Jan was the most connected author.

The citation impact of the different collaboration patterns among countries and institutions was analysed by one-way ANOVA, and it was statistically significant (P < 0.001) (Table 2). The highest citation impact was observed for a single country’s publication and three institutions' collaboration.

**Table 2:** Collaboration patterns of countries and institution *P < 0.05: statistically significant

Collaboration patterns by countries	Total publications	Total citations	Citation impact	p-value
Single country’s publications	60	14794	246.57	<0.001
Two countries’ publications	22	4290	195.00	
Three countries' publications	11	2428	220.73	
More than three countries' publications	7	1307	186.71	
Collaboration patterns by institutions				
Single Institutions	22	4167	189.41	<0.001
Two institutions	28	5968	213.14	
Three institutions	15	5137	342.47	
Four institutions	13	2629	202.23	
Five institutions	7	1632	233.14	
More than five institutions	15	3286	219.07	

Keyword Analysis

**Figure 5:**
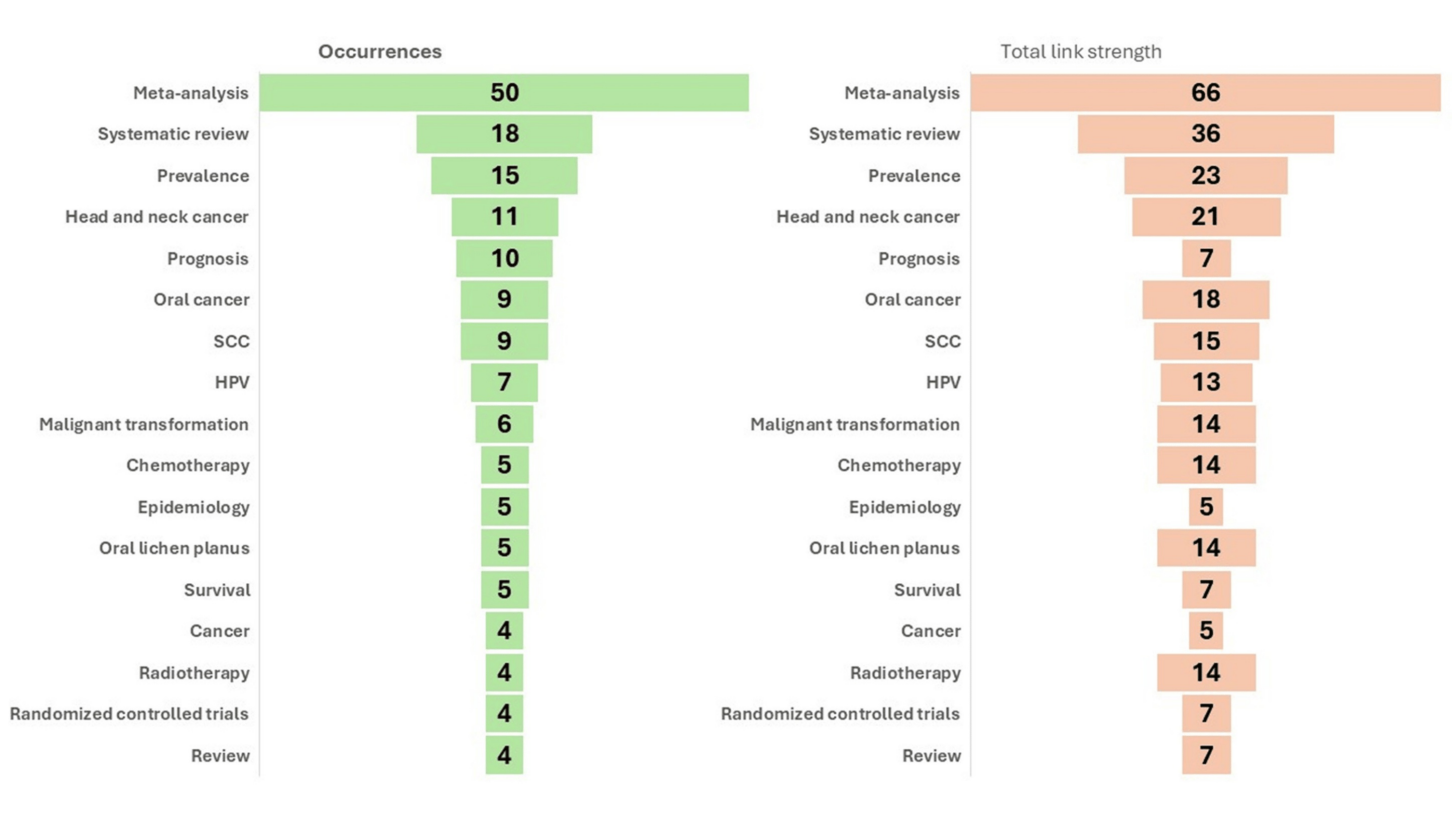
Seventeen most occurred keywords and their cumulative link strength

A total of 263 keywords had been used by the authors, and 80.22% (n = 211) keywords were used one time. The top 17 keywords are shown in Figure 5 with occurrences in the dataset and the total link strength associated with each keyword. The keyword “meta-analysis” appeared most frequently in terms of occurrence and total link strength. This suggests that "meta-analysis" is a central topic in the dataset, and it is highly interconnected with other terms. The keyword "systematic review" ranks second, followed by "prevalence". The dataset focused on cancer research, particularly oral and head/neck cancers and treatments like chemotherapy and radiotherapy. The prominence of terms related to prognosis, survival, and malignant transformation highlights the focus on cancer progression and outcomes. Keywords related to epidemiology and randomised controlled trials suggest that these studies could involve SR of prospective studies and distribution patterns in cancer research.

**Figure 6:**
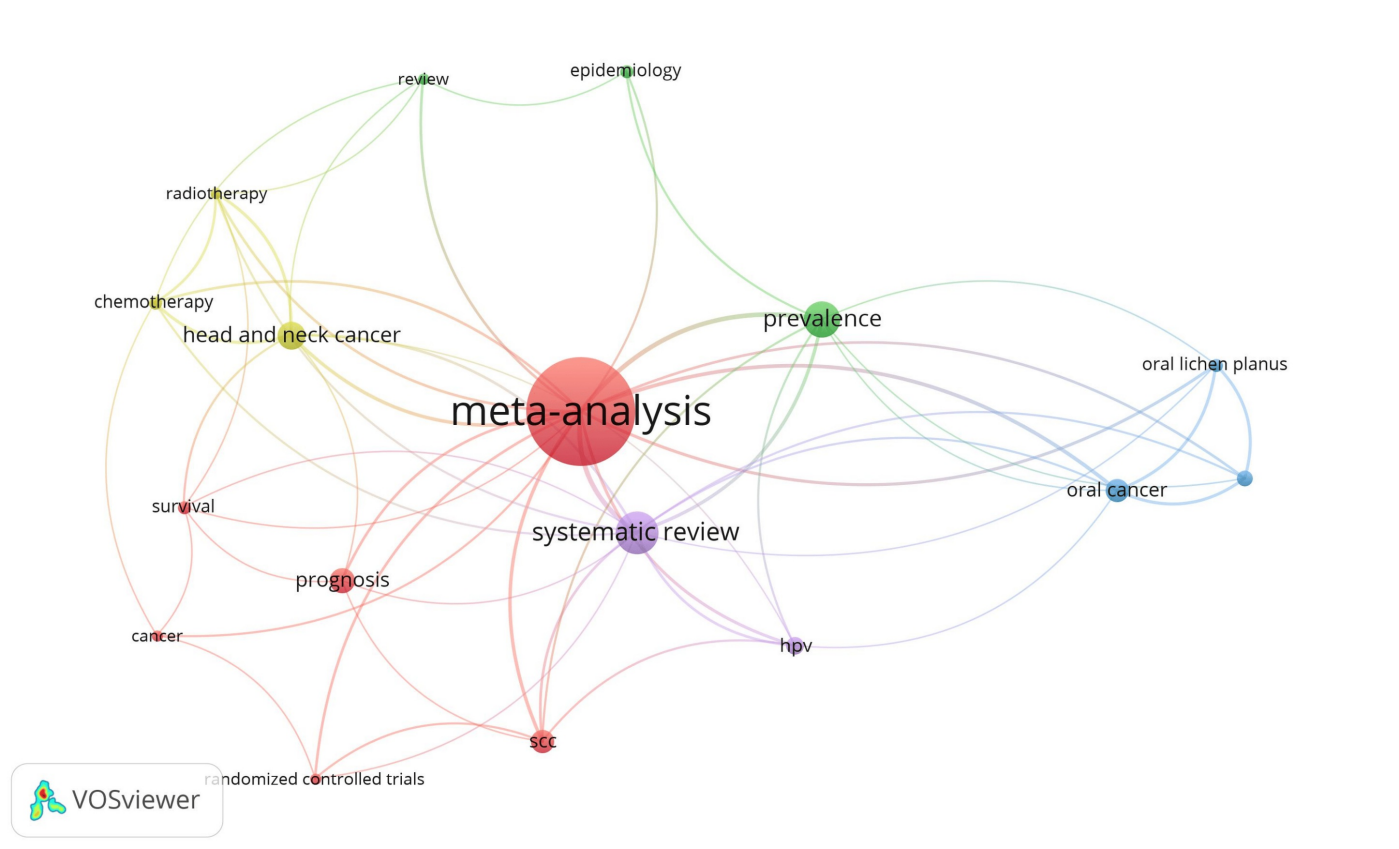
Co-occurrence network of the top keywords

A keyword co-occurrence network was done with the help of Vosviewer software, as shown in Figure 6. The clustering method identifies keywords that often interact or link with one another and aggregates them into a cluster. The top 17 most occurred keywords have been divided into five coloured clusters: Red Cluster (six keywords): cancer, meta-analysis, prognosis, randomized controlled trials, SCC, survival; Green Cluster (three keywords): epidemiology, prevalence, review; Blue Cluster (three keywords): malignant transformation, oral cancer, oral lichen planus; Yellow Cluster (three keywords): chemotherapy, head and neck cancer, radiotherapy; Purple Cluster (two keywords): HPV, systematic review.

Articles Based on Research Domains and Characteristics of the 100 Most Cited Articles

**Figure 7:**
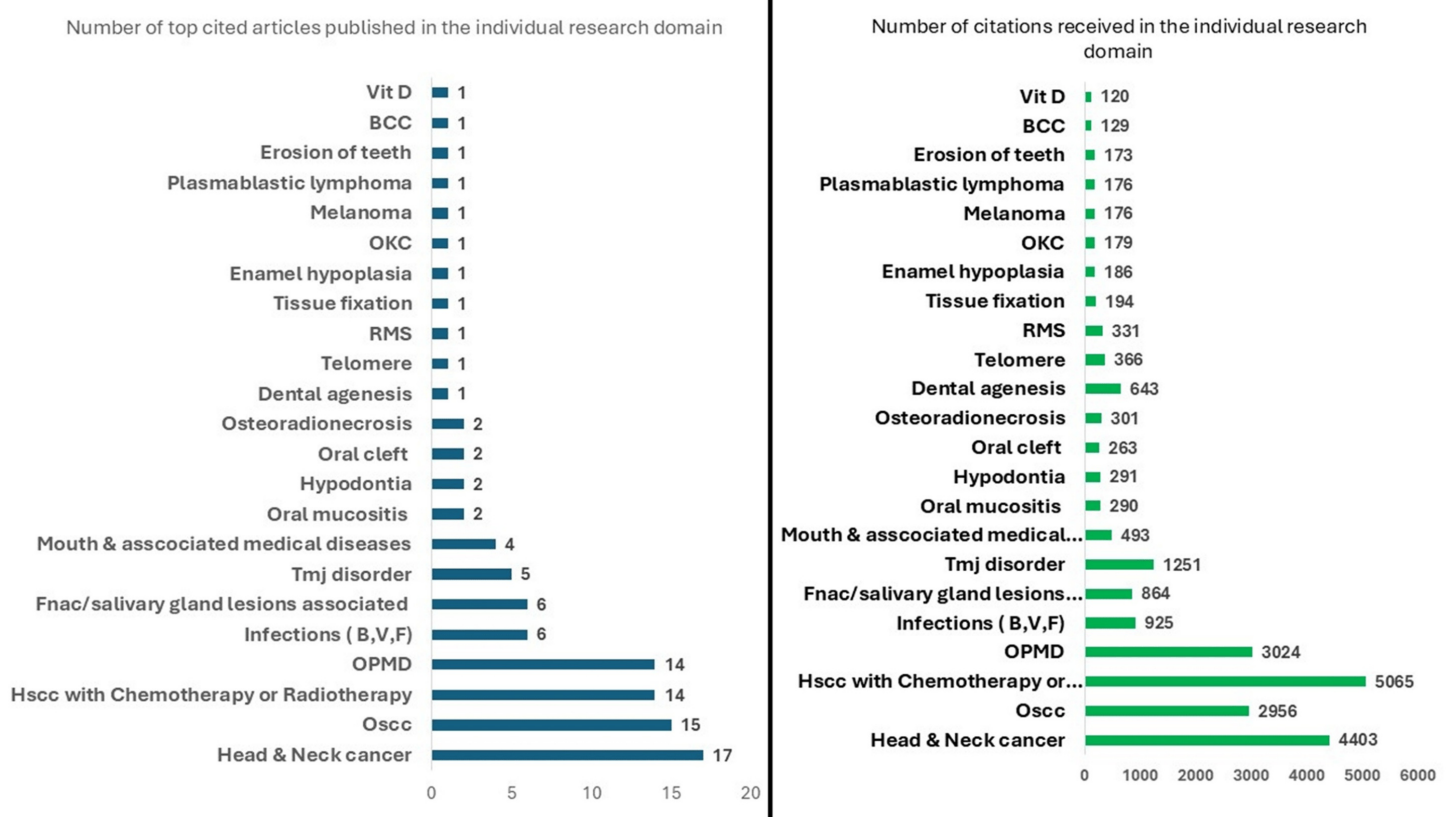
Allocation of publications according to the research area and number of citations BCC: basal cell carcinoma, RMS: rhabdomyosarcoma, OKC: odontogenic keratocyst, OPMD: oral potentially malignant disorders, (B, V, F): bacterial, viral, fungal, OSCC: oral squamous cell carcinoma

Twenty-three areas were examined among the top 100 cited SRs and MAs. Figure 7 shows 23 research domains with respect to the number of publications and citations. The primary areas of focus were four research domains, which were head and neck cancer, oral squamous cell carcinoma, head and neck cancer with chemotherapy or radiotherapy, and oral potentially malignant disorders. It can be clearly seen that with recent advances in diagnostic techniques and IHC markers, the area of research in oral and head/ neck cancers has significantly increased in the past years and is directly proportional to the significant growth of publications and citations received. Research areas on basal cell carcinoma, plasmablastic lymphomas, and the overall received and least number of publications with citations.

The citation impact among subject specialities, type of review articles, and journal types was done using the Pearson Chi-square test, and it was statistically significant (P < 0.001) (Table 3). The citation impact of *Head and Neck Pathology*, SRMA, and non-dental journals was higher than their counterparts. The appendix includes the list of the 100 most cited referenced articles, along with citation density by rank.

**Table 3:** Distribution of articles categorized by subject speciality, type of reviews, and type of journals *P < 0.05: statistically significant

Subject speciality	Total publications	Total citations	Citation impact	p-value
Oral pathology /oral med	63	11968	189.96	<0.001
Head and neck pathology	37	10851	293.27	
Type of review articles				
Systematic review only	16	3193	199.56	<0.001
Systematic review and meta-analysis	84	19626	233.64	
Type of journals				
1. Dental journals	45	8875	197.22	<0.001
2. Non-dental journals	55	13944	253.52	

Discussion

Bibliometric analysis assesses publication and citation trends to measure the significance, impact, and influence of research within a certain topic. It also assists academicians and organisations in determining topics for future research or collaboration [10]. Bibliometric analysis on SRs and MAs on the 100 most cited articles has been done on topics like dentistry [11], orthodontics [12], and oral surgery [13]. To our knowledge, no bibliometric study solely focused on SRs and MAs in oral pathology has been undertaken to date.

Since oral medicine and oral pathology are two interrelated dental specialities focused on the diagnosis and non-surgical treatment of oral problems, as well as the oral healthcare of patients with chronic, recurring, and medically associated conditions of the oral and maxillofacial area were considered. Among the top cited 100 articles in the present review, 63 articles related to oral pathology and 37 articles related to head and neck pathology were found. This study sought to delineate the scientometric landscape of SR and MA publications in the field of oral pathology from the year 1990 to 2022. The data indicate a progressive rise in annual output within the field of oral pathology overall, as well as in most of its associated research areas in the head and neck cancer field. The United States and England emerged as the most productive nations, while France distinguished itself by hosting the most prolific and influential scholars and institutions. The variations in publishing output among nations can be attributed to multiple factors, including the size of the scientific population, the vibrancy of the research community, access to online databases, funding for research-related capital projects, and proficiency in the English language. The selection of keywords utilised for data inquiries is one of the elements that may influence the substantial quantity and quality of biomedical literature [6]. Bibliometric findings of Western countries being the most productive nations have also been observed in numerous bibliometric studies [6,7,8,10]. The reasons could be due to the size of the scientific population, the presence of an active research community, access to online databases, funding for research-related capital projects, proficiency in English, and the choice of keywords used in data searches. The engagement in international collaboration among prominent authors was notably vigorous [6]. 

The body of scientific literature pertaining to oral pathology is extensive. Reports indicate that fewer than 10% of research papers attain the distinction of classic articles; also, an article that has acquired 100 or more citations is often considered a 'classic' within its study domain [14]. In extensive research domains, publications that exceed 400 citations are also regarded as classics. Nonetheless, the traditional citation differs across various disciplines [15]. In the current review, all the top 100 cited articles surpassed more than 100 citations, while seven articles crossed more than 400 citations and were thus referred to as classified or seminal work. The elevated proportion is to be expected, as numerous publications relate to the latest advancements in the field, reflecting a surge in research and publications, alongside the inclination of authors to preferentially cite articles that exhibit higher levels of evidence [16].

Overall, the SRs and MAs had covered 23 subject domains with head and neck cancer, oral cancer, and oral potentially malignant disorders topping the list with 15,448 total citations from 60 articles. This finding is unsurprising, as it represents a core area within the field of oral pathology. It is only natural to observe a rise in the number of citations of landmark publications in a given domain, as recent advancements in any discipline demonstrate a greater knowledge gap, elicit a greater level of interest, and encourage additional research and publication [17]. Among the distinct individual articles, the top most cited article belonged to the head and neck cancer category, which got the top citations of 2,228 published in the journal of radiotherapy oncology, while among the oral pathology field, the top citation was in oral squamous cell carcinoma, which received 458 citations published by journal of* Oral Surgery, Oral Medicine, Oral Pathology, and Oral Radiology*. It is important to note that 25% of the publications were published in journals not only dedicated to the field of oral pathology, underscoring the necessity to explore outside speciality journals while seeking pertinent information. A similar distribution of varied journal options was seen in a study done by Selvaraj et al. [12].

The journal metrics included in the present review were the cite score, H index, and JIF. The cite score metric of Scopus is calculated by comparing the average number of citations received per document in a particular year to the total number of articles published in the previous three years [18]. JIF is a metric that quantifies the average frequency with which articles in a journal are cited. The data are collected in WoS JCR (journal citation reports), which provides a catalogue of journals and their impact factors [19]. Hirsch's h-index quantifies the aggregate number of citations generated by an author's publications by analysing the most frequently cited articles. It denotes the total number of papers (h) that have received at least 'h' citations by an author. These indicators serve as metrics for scholarly output and the influence of scientific contributions [19]. In the present review, the citation indicators like JIF (average: 12.04), Scopus cite score (average: 18.3), and H index (average: 3.48) for the top cited 17 journals were recorded.

The duration since publication is a crucial element that influences the citation metrics of an article. Older papers gain more citations than the new ones, irrespective of their significance. This inclination is seen in nearly all disciplines [20,21]. Kuhnian philosophy believes that scientific communities exhibit a tendency to adhere to established patterns, potentially resulting in a 'snowball effect' in citations, as authors prefer to cite publications that are already widely referenced rather than critically evaluating their relevance and quality [22]. Consistent with earlier research, more cited publications were published post-2010 [13,23], underscoring the scientific advancement in oral pathology centred on clinical trials and evidence-based practice.

Keywords are essential since they yield more pertinent results during literature searches compared to sentences or phrases. Keywords serve several purposes; primarily, they encapsulate three to five essential terms inside an author's work. Secondly, to find prevalent academic themes from both historical and contemporary contexts by keyword analysis. Thirdly, a paper's likelihood of being cited increases with the presence of certain associated keywords [24]. This study employed VOSviewer to create a map of co-authors and keywords derived from co-occurrence data. The distance between two items indicates the strength of their link; a shorter distance signifies a greater connection. Items are often dispersed throughout distance-based maps, facilitating the identification of clusters of related objects. Conversely, it can sometimes complicate the accurate labelling of each item on a map without overlap. The clustering technique was employed to assign a country and keywords denoted by colour [7]. The size of the circles in VOSviewer indicates keyword occurrences; the wider the circle, the more keywords were co-selected. The distance between the two keywords revealed relative strength and topic similarity. Circles in the same colour cluster indicate a common theme across these articles, whilst links indicate a co-occurrence relationship between two terms. According to the VOSviewer handbook, each link has a strength, which is represented by a positive number, i.e., the greater the number, the more powerful the relationship. The total link strength reflects the number of articles that contain two keywords together [25]. In the present review, the top keywords, VOSviewer software, occurrences, and total link strength revealed that the most common keywords used were "meta-analysis" and "systematic review". The same two keywords were the most occurred in another bibliometric study on SRs and MAs in endodontics [23].

In the present review, WoS was the main citation database used. The WoS has historically established the criteria for counting citations. The WoS asserts that a key strength lies in its selective approach to selecting certain publications in its content coverage. Bradford’s Law, introduced in 1934, posits that a significant proportion of major scientific discoveries is disseminated through a limited number of journals. Consequently, the WoS prioritises the quality of its content coverage over quantity [26]. It is frequently utilised in bibliometric analyses because of its extensive subject scope and the capability to quantify citations of articles and evaluate the contributing institutions for each study [11]. Nevertheless, WoS continues to be the most prominent and extensively employed database for citation analysis across all academic fields [12]. Moreover, PubMed was selected because of its status as the most authoritative and extensively utilised biomedical database, guaranteeing the inclusion of high-quality, peer-reviewed literature in the health sciences. Our emphasis on SRs and MAs in oral pathology ensured that PubMed's stringent indexing criteria upheld the trustworthiness and scientific integrity of the selected articles. Although other databases may have a broader range, PubMed was selected to ensure specificity, trustworthiness, and pertinence to biological and clinical research.

Ultimately arriving at the key takeaway, the significance of SRs and MAs in healthcare has been escalating. Clinicians peruse such publications to remain updated in their profession; they also serve as a foundation for formulating clinical practice recommendations [27]. When methodologically conducted, SRs and MAs can produce robust outcomes often attainable only through extensive randomised controlled trials, which are challenging to execute in individual research [5]. Consequently, it is crucial to utilise the findings of this article as a resource for researchers, particularly in areas that significantly influence the field of oral & head neck pathology.

Limitations

The present bibliometric study exclusively utilised the WoS database, potentially excluding pertinent publications not indexed by WoS. Additional databases, like Scopus and Google Scholar, include bibliometric information on published publications. The content scope of Google Scholar is unclear, and the reliability of results is variable, whilst citation analysis is neglected by PubMed. Google Scholar has faced criticism, partly for using citations from sources deemed non-scholarly, such as student handbooks and administrative notes. Google Scholar utilises automatic web crawlers to collect information from internet content; however, the technique employed to connect records remains undisclosed [26]. Google Scholar does not provide a list of included journal titles or coverage dates; nevertheless, it has stated that it has partnerships with most major publishers. Google Scholar does not specify the criteria for a website's inclusion in its search results [28]. Scopus has constraints regarding its coverage of earlier publications. However, citation coverage only extends back to 1996. While abstracts are available for some journals dating back to 1966, the lack of citation coverage from that earlier period presents a limitation for conducting citation analysis. Scopus features its own web search engine, Scirus. The results from Scirus are displayed independently of the results of other Scopus journals. In addition, material sourced from Scirus is not included in the citation counts for Scopus journal records [28].

The present review may have overlooked articles published in non-indexed and non-English journals. The number of citations determines the impact and quality of an article; regrettably, it may be contingent upon time. A further limitation is the possible mistake introduced in bibliometric studies due to 'self-citation' and 'journal bias'. The former suggests the authors' inclination to reference their own articles to enhance their credentials and the journal's impact factor. Journal bias denotes the tendency of authors to reference articles from the same journal to which they aspire to submit their study [12]. Further citation metrics must not be construed as direct measures of research quality. Elevated citation counts do not inherently signify robust or impactful research. Finally, the quality assessment of top-cited SRs and MAs, critical appraisals, and heterogeneity in study design, along with the translation value of the research, have not been evaluated and are outside the parameters of this study. Further research is necessary to conduct such analyses centred on a specific subject and also to amalgamate all search databases for comprehensive coverage of this extensive subject.

## Conclusions

This review presents the first comprehensive bibliometric analysis of SRs and MAs on oral pathology from 1990 to 2022. The 100 most cited articles were published across 67 dental journals and 33 non-dental journals, indicating a multidisciplinary interest in the field. The articles frequently featured multiple authors and encompassed collaborative efforts from various universities. The focus of the research was centred on head and neck cancer with chemotherapy and radiotherapy, oral cancer, and oral potentially malignant disorders. There has been an increasing trend in the publication of articles on this topic since 2009, underscoring the significance of the topic. This review serves as a valuable resource for researchers and clinicians in the fields of oral medicine, oral pathology, head and neck oncology, and maxillofacial surgery. Future research is necessary, as science is continually evolving, and the compilation of newly published articles requires regular updates.

## Appendices

**Table 4:** Appendices (100 most cited articles with citation density by rank)

Serial No.	Details of articles	Total citations	Citation density by year (rank)
1.	Meta-analysis of chemotherapy in head and neck cancer (MACH-NC): an update on 93 randomised trials and 17,346 patients [29]	2228	139.25 (1)
2.	A meta-analysis of the prevalence of dental agenesis of permanent teeth [30]	643	30.62 (17)
3.	Prevalence of human papillomavirus in oropharyngeal and nonoropharyngeal head and neck cancer-systematic review and meta-analysis of trends by time and region [31]	584	48.67 (6)
4.	Survival of squamous cell carcinoma of the head and neck in relation to human papillomavirus infection: review and meta-analysis [32]	557	30.94 (16)
5.	HPV DNA, E6/E7 mRNA, and p16INK4a detection in head and neck cancers: a systematic review and meta-analysis [33]	545	49.55 (5)
6.	Oral squamous cell carcinoma: review of prognostic and predictive factors [34]	458	24.11 (32)
7.	Meta-analysis of chemotherapy in head and neck cancer (MACH-NC): a comprehensive analysis by tumour site [35]	440	31.43 (15)
8.	Meta-analysis of the impact of human papillomavirus (HPV) on cancer risk and overall survival in head and neck squamous cell carcinomas (HNSCC) [36]	370	24.67 (30)
9.	The association of telomere length and cancer: a meta-analysis [37]	366	26.14 (26)
10.	Hypoxic modification of radiotherapy in squamous cell carcinoma of the head and neck--a systematic review and meta-analysis [38]	358	25.57 (28)
11.	Prevalence of temporomandibular joint disorders: a systematic review and meta-analysis [39]	357	89.25 (2)
12.	Fusion gene-negative alveolar rhabdomyosarcoma is clinically and molecularly indistinguishable from embryonal rhabdomyosarcoma [40]	331	22.07 (34)
13.	Human papillomavirus as a risk factor for oral squamous cell carcinoma: a meta-analysis, 1982-1997 [41]	328	13.67 (63)
14.	Oral epithelial dysplasia and the development of invasive squamous cell carcinoma [42]	328	10.93 (73)
15.	Human papillomavirus-related head and neck cancer survival: a systematic review and meta-analysis [43]	315	24.23 (31)
16.	Relationship between bruxism and temporomandibular disorders: a systematic review of literature from 1998 to 2008 [44]	304	20.27 (36)
17.	Adjuvant and adjunctive chemotherapy in the management of squamous cell carcinoma of the head and neck region. A meta-analysis of prospective and randomized trials [45]	301	10.38 (75)
18.	Malignant transformation of oral leukoplakia: a systematic review of observational studies [46]	283	31.44 (14)
19.	Lifelong Learning Programme. An appraisal of oral cancer and pre-cancer screening programmes in Europe: a systematic review [47]	283	28.30 (21)
20.	Predictive value of tumor thickness for cervical lymph-node involvement in squamous cell carcinoma of the oral cavity: a meta-analysis of reported studies [48]	282	17.63 (43)
21.	Treatment and follow-up of oral dysplasia - a systematic review and meta-analysis [49]	279	17.44 (44)
22.	An overview of randomised controlled trials of adjuvant chemotherapy in head and neck cancer [50]	279	9.30 (83)
23.	Potentially malignant disorders of the oral cavity and oral dysplasia: A systematic review and meta-analysis of malignant transformation rate by subtype [51]	267	53.40 (3)
24.	Socioeconomic inequalities and oral cancer risk: a systematic review and meta-analysis of case-control studies [52]	248	14.59 (57)
25.	Gender differences in temporomandibular disorders in adult populational studies: A systematic review and meta-analysis [53]	238	34.00 (10)
26.	The prognostic role of tumor infiltrating T-lymphocytes in squamous cell carcinoma of the head and neck: A systematic review and meta-analysis [54]	235	29.38 (18)
27.	Human papillomaviruses in oral carcinoma and oral potentially malignant disorders: a systematic review [55]	235	16.79 (48)
28.	Oral acyclovir therapy accelerates pain resolution in patients with herpes zoster: a meta-analysis of placebo-controlled trials [56]	234	8.07 (88)
29.	Correlation between apparent diffusion coefficient (ADC) and cellularity is different in several tumors: a meta-analysis [57]	233	29.13 (20)
30.	A meta-analysis of hyper fractionated and accelerated radiotherapy and combined chemotherapy and radiotherapy regimens in unresected locally advanced squamous cell carcinoma of the head and neck [58]	225	11.84 (69)
31.	Prevalence of oral potentially malignant disorders: A systematic review and meta-analysis [59]	222	31.71 (12)
32.	A systematic review and meta-analysis of the diagnostic accuracy of fine-needle aspiration cytology for parotid gland lesions [60]	222	15.86 (51)
33.	HPV in oral squamous cell carcinoma vs head and neck squamous cell carcinoma biopsies: a meta-analysis (1988-2007) [61]	216	12.71 (66)
34.	Taxane-cisplatin-fluorouracil as induction chemotherapy in locally advanced head and neck cancers: an individual patient data meta-analysis of the meta-analysis of chemotherapy in head and neck cancer group [62]	215	17.92 (42)
35.	Prevalence in the Dutch adult population and a meta-analysis of signs and symptoms of temporomandibular disorder [63]	212	6.63 (94)
36.	Role of radiotherapy fractionation in head and neck cancers (MARCH): an updated meta-analysis [64]	208	26.00 (27)
37.	Intensity-modulated radiation therapy for head and neck cancer: systematic review and meta-analysis [65]	204	18.55 (41)
38.	Worldwide prevalence of oral lichen planus: A systematic review and meta-analysis [66]	201	50.25 (4)
39.	Prognostic significance of vascular endothelial growth factor immunohistochemical expression in head and neck squamous cell carcinoma: a meta-analysis [67]	196	9.80 (77)
40.	Effects of preanalytical variables on the detection of proteins by immunohistochemistry in formalin-fixed, paraffin-embedded tissue [68]	194	13.86 (61)
41.	Global burden of molar incisor hypomineralization [69]	186	26.57 (25)
42.	Diagnostic accuracy of p16^INK4a^ immunohistochemistry in oropharyngeal squamous cell carcinomas: A systematic review and meta-analysis [70]	185	23.13 (33)
43.	Meta-analysis of chemotherapy in head and neck cancer (MACH-NC): An update on 107 randomized trials and 19,805 patients, on behalf of MACH-NC Group [71]	180	45.00 (7)
44.	Risk factors for osteoradionecrosis after head and neck radiation: a systematic review [72]	179	13.77 (62)
45.	Systematic review of the treatment and prognosis of the odontogenic keratocyst [73]	179	7.16 (92)
46.	Meta- and pooled analyses of GSTM1, GSTT1, GSTP1, and CYP1A1 genotypes and risk of head and neck cancer [74]	178	8.09 (87)
47.	Prognostic value of tumor-infiltrating lymphocytes in melanoma: a systematic review and meta-analysis [75]	176	29.33 (19)
48.	Malignant transformation of oral lichen planus and oral lichenoid lesions: A meta-analysis of 20095 patient data [76]	176	22.00 (35)
49.	Clinicopathologic comparison of plasmablastic lymphoma in HIV-positive, immunocompetent, and posttransplant patients: single-center series of 25 cases and meta-analysis of 277 reported cases [77]	176	16.00 (50)
50.	Estimated prevalence of erosive tooth wear in permanent teeth of children and adolescents: an epidemiological systematic review and meta-regression analysis [78]	173	17.30 (45)
51.	A systematic review with meta-analysis of the effect of low-level laser therapy (LLLT) in cancer therapy-induced oral mucositis [79]	173	12.36 (68)
52.	Human papillomavirus expression in oral mucosa, premalignant conditions, and squamous cell carcinoma: a retrospective review of the literature [80]	173	5.97 (98)
53.	Osteopontin is a marker for cancer aggressiveness and patient survival [81]	170	11.33 (71)
54.	Detection of cervical lymph node metastasis in head and neck cancer patients with clinically N0 neck-a meta-analysis comparing different imaging modalities [82]	163	12.54 (67)
55.	The reported prevalence of oral lichen planus: a review and critique [83]	163	9.59 (81)
56.	Malignant transformation risk of oral lichen planus: A systematic review and comprehensive meta-analysis [84]	160	26.67 (24)
57.	Prevalence of hypodontia and associated factors: a systematic review and meta-analysis [85]	158	14.36 (59)
58.	Rate of malignant transformation of oral lichen planus: A systematic review [86]	153	25.50 (29)
59.	Prognostic biomarkers for oral tongue squamous cell carcinoma: a systematic review and meta-analysis [87]	153	19.13 (40)
60.	Extracapsular spread in head and neck squamous cell carcinoma: A systematic review and meta-analysis [88]	153	17.00 (47)
61.	Oral Manifestations in Patients with COVID-19: A Living Systematic Review [89]	151	37.75 (9)
62.	Significance of CD44 expression in head and neck cancer: a systemic review and meta-analysis [90]	150	13.64 (64)
63.	Betel quid chewing and the risk of oral and oropharyngeal cancers: a meta-analysis with implications for cancer control [91]	144	13.09 (65)
64.	Maternal cigarette smoking and oral clefts: a meta-analysis [92]	144	5.14 (99)
65.	Prognostic role of pretreatment plasma fibrinogen in patients with solid tumors: A systematic review and meta-analysis [93]	142	14.20 (60)
66.	Role of ultrasound-guided core-needle biopsy in the assessment of head and neck lesions: a meta-analysis and systematic review of the literature [94]	141	10.85 (74)
67.	The hierarchy of different treatments for arthrogenous temporomandibular disorders: A network meta-analysis of randomized clinical trials [95]	140	28.00 (22)
68.	Reliability of HIV rapid diagnostic tests for self-testing compared with testing by health-care workers: a systematic review and meta-analysis [96]	140	20.00 (38)
69.	Hepatitis C virus infection and lichen planus: a systematic review with meta-analysis [97]	140	9.33 (82)
70.	Risk Factors for Herpes Zoster Infection: A Meta-Analysis [98]	137	27.40 (23)
71.	Candida albicans and Early Childhood Caries: A Systematic Review and Meta-Analysis [99]	137	19.57 (39)
72.	A meta-analysis of the randomized controlled trials on elective neck dissection versus therapeutic neck dissection in oral cavity cancers with clinically node-negative neck [100]	135	9.64 (79)
73.	Malignant transformation of oral leukoplakia: Systematic review and meta-analysis of the last 5 years [101]	134	33.50 (11)
74.	Sensitivity, Specificity, and Posttest Probability of Parotid Fine-Needle Aspiration: A Systematic Review and Meta-analysis [102]	134	14.89 (55)
75.	Has hypodontia increased in Caucasians during the 20th century? A meta-analysis [103]	133	6.33 (95)
76.	Is diagnostic delay related to advanced-stage oral cancer? A meta-analysis [104]	132	8.25 (86)
77.	Quality of life in survivors of oropharyngeal cancer: A systematic review and meta-analysis of 1366 patients [105]	131	16.38 (49)
78.	Elective Neck Dissection vs Observation in Early-Stage Squamous Cell Carcinoma of the Oral Tongue With No Clinically Apparent Lymph Node Metastasis in the Neck: A Systematic Review and Meta-analysis [106]	131	14.56 (58)
79.	Adjuvant chemotherapy in head and neck cancer [107]	131	3.74 (100)
80.	What is the best surgical margin for a Basal cell carcinoma: a meta-analysis of the literature [108]	129	8.60 (85)
81.	Surrogate endpoints for overall survival in locally advanced head and neck cancer: meta-analyses of individual patient data [109]	128	8.00 (89)
82.	Post-COVID syndrome. A case series and comprehensive review [110]	126	31.50 (13)
83.	HLA and Sjögren's syndrome susceptibility. A meta-analysis of worldwide studies [111]	126	9.69 (78)
84.	Fine-needle aspiration cytology in a regional head and neck cancer center: comparison with a systematic review and meta-analysis [112]	126	7.41 (91)
85.	Oral manifestations of Diabetes Mellitus. A systematic review [113]	125	15.63 (52)
86.	Xerostomia and salivary hypofunction in vulnerable elders: prevalence and etiology [114]	125	9.62 (80)
87.	Periodontal disease, edentulism, and pancreatic cancer: a meta-analysis [115]	124	15.50 (53)
88.	Meta-analysis of dysphagia and aspiration pneumonia in frail elders [116]	123	8.79 (84)
89.	Risk of osteonecrosis of the jaw in cancer patients receiving denosumab: a meta-analysis of seven randomized controlled trials [117]	122	11.09 (72)
90.	Individual patients' data meta-analyses in head and neck cancer [118]	122	6.78 (93)
91.	Evidence-based clinical practice guideline on antibiotic use for the urgent management of pulpal- and periapical-related dental pain and intraoral swelling: A report from the American Dental Association [119]	121	20.17 (37)
92.	Sentinel node biopsy for squamous cell carcinoma of the oral cavity and oropharynx: a diagnostic meta-analysis [120]	121	10.08 (76)
93.	The impact of vitamin D pathway genetic variation and circulating 25-hydroxyvitamin D on cancer outcome: systematic review and meta-analysis [121]	120	15.00 (54)
94.	Global prevalence of cleft palate, cleft lip and cleft palate and lip: A comprehensive systematic review and meta-analysis [122]	119	39.67 (8)
95.	Tumour budding in oral squamous cell carcinoma: a meta-analysis [123]	119	17.00 (46)
96.	Weekly Low-Dose Versus Three-Weekly High-Dose Cisplatin for Concurrent Chemoradiation in Locoregionally Advanced Non-Nasopharyngeal Head and Neck Cancer: A Systematic Review and Meta-Analysis of Aggregate Data [124]	118	14.75 (56)
97.	MicroRNAs as prognostic molecular signatures in human head and neck squamous cell carcinoma: a systematic review and meta-analysis [125]	118	11.80 (70)
98.	Preventive intervention possibilities in radiotherapy- and chemotherapy-induced oral mucositis: results of meta-analyses [126]	117	6.16 (96)
99.	Effects of antioxidant supplements on cancer prevention: meta-analysis of randomized controlled trials [127]	116	7.73 (90)
100.	Drooling of saliva: a review of the etiology and management options [128]	116	6.11 (97)
